# Health-related quality of life in patients with polycystic ovary syndrome: validation of the German PCOSQ-G

**DOI:** 10.1007/s00404-017-4623-2

**Published:** 2017-12-16

**Authors:** B. Böttcher, S. Fessler, F. Friedl, B. Toth, M. H. Walter, L. Wildt, D. Riedl

**Affiliations:** 1 Department of Gynecological Endocrinology and Reproductive Medicine, Medical University Innsbruck, Anichstrasse 35, 6020 Innsbruck, Austria; 2 Department of Psychology, University of Innsbruck, Bruno-Sander-Haus, Innrain 52f, 6020 Innsbruck, Austria; 3 University Clinic of Medical Psychology, Medical University Innsbruck, Schöpfstr. 23a, 6020 Innsbruck, Austria

**Keywords:** Polycystic ovary syndrome, Quality of life, Anxiety, Depression, PCOSQ, PCOSQ-G validation

## Abstract

**Purpose:**

Patients with polycystic ovary syndrome (PCOS) report a decreased health-related quality of life (HRQOL) and higher levels of psychological distress. Validated questionnaires are necessary to assess the impact of PCOS on patients’ lives. The aim of the present study was to evaluate the German “Polycystic Ovary Syndrome Questionnaire” (PCOSQ-G).

**Methods:**

The psychometric properties of the PCOSQ-G were investigated in PCOS patients with item-total correlation, internal consistency and test–retest reliability. Correlations with the Short-Form-36 Health Survey (SF-36) and the Hospital Anxiety and Depression Scale (HADS-D) were calculated to evaluate the validity of the PCOSQ-G. Discriminatory validity was investigated through a receiver operating characteristic curve and independent sample *t* tests compared with healthy controls.

**Results:**

Good psychometric properties were found for most items. Acceptable to high internal consistency was found for the total score (*α* = 0.94–0.95) and all subscales (*α* = 0.70–0.97). High test–retest reliability was found for the total score (0.86) and all subscales (0.81–0.90). The validity analyses showed that the PCOSQ-G total score was positively correlated with both SF-36 summary scales and was negatively correlated with both HADS subscales. Patients reported significantly lower values for the PCOSQ-G total score (*p* < 0.001) and all subscales, and the PCOSQ-G discriminated well between patients and healthy controls (AUC = 0.81, *p* < 0.001).

**Conclusions:**

PCOSQ-G is a reliable and valid tool to assess the HRQOL in patients with PCOS and can be used in future clinical research. Patients with PCOS exhibited an impaired HRQOL, which indicates the need for psychosomatic counseling.

**Electronic supplementary material:**

The online version of this article (10.1007/s00404-017-4623-2) contains supplementary material, which is available to authorized users.

## Introduction

Polycystic ovary syndrome (PCOS) is a common endocrine disorder that affects up to 12% of women of reproductive age [1–3]. Following the Rotterdam criteria, PCOS is characterized by oligo- or anovulation, clinical or biochemical aspects of hyperandrogenism and the presence of polycystic ovaries on ultrasound [4]. A growing body of evidence suggests that impaired mental health and a reduced health-related quality of life (HRQOL) may be significantly associated with this disorder [5, 6]. More specifically, several studies in different patient populations and meta-analyses of these studies have shown that women with PCOS have an increased risk of anxiety and depression symptoms [5, 7–17] and a reduced HRQOL [18–23]. These scores are higher than those of healthy controls, but nevertheless are usually within the normal range [7, 8, 12]. Additionally, in the current studies, the percentage ranges of women affected by anxiety and depression are extremely variable, especially when subgroup analyses are performed based on the body mass index and clinical signs of hyperandrogenism [24–30].

Due to these conflicting results, we aimed to examine our own patient cohort with PCOS and hypothesized that our patients would show higher depression and anxiety scores and a reduced HRQOL.

While generic instruments to assess HRQOL allow to compare the impairing effects of different physical diseases, they do not cover all central areas of health and well-being of PCOS. The health-related quality of life questionnaire for women with PCOS (PCOSQ) is a disease-specific, validated questionnaire developed by Cronin et al. [31]. The PCOSQ consists of 26 items that are categorized into five domains after factor analysis (emotion, hirsutism, weight, infertility, and menstruation). The questionnaire takes 10–15 min to complete. To date, the questionnaire has not been used in German-speaking countries. Its distinctive feature is the revelation of disease-specific impairments and symptoms like hirsutism, infertility or menstrual irregularities which occur in PCOS patients.

The main objective of this study was to validate a German version of the PCOSQ and to confirm its reliability to facilitate further studies in German-speaking populations.

## Methods

### Patients and procedures

Patients (*n* = 199) aged between 18 and 45 years who were diagnosed with PCOS were recruited from the outpatient department of the University Clinic of Gynecologic Endocrinology and Reproductive Medicine Innsbruck, Austria, between 2012 and 2014. The medical history and sociodemographic data were collected in personal interviews and with self-administered questionnaires. PCOS was diagnosed according to the Rotterdam criteria [4]. No specific exclusion criteria except for scarce German language skills were applied. A healthy control sample of women within the same age group recruited in a student cohort via internet had the option to return an online version of the questionnaire. The patients and controls received a questionnaire battery that took approximately 40 min to complete. The patients received the PCOSQ again after 4 weeks for validation of the questionnaire.

The study was conducted in accordance with the Declaration of Helsinki. The Ethics Committee of the Medical University Innsbruck approved the study design (AN-5233-329/4.14). Written informed consent was obtained from all participants.

### Assessment instruments

#### Anxiety and depression

The German version of the Hospital Anxiety and Depression Scale (HADS) [32, 33] was used to assess anxiety and depression. The questionnaire is designed for patients with somatic diseases and aims to identify typical aspects of anxiety and depression within the previous 7 days. It consists of 14 items and can be divided into two subscales (anxiety and depression) with seven items each. The items can also be summed to a total score, with higher scores indicating larger distress. Values of 0–7, 8–10, and > 11 in the anxiety subscale and 0–5, 6–8, and > 9 in the depression subscale are classified as non-cases, probable cases, and cases, respectively [32].

#### Health-related quality of life

General HRQOL was assessed using the German Version of the Short-Form-36 Health Survey (SF-36) [34, 35]. The questionnaire consists of 36 items containing eight subscales each. These subscales constitute two higher-level summary scales [the physical (physical function, physical role function, bodily pain, and general health) and psychological (vitality, social function, emotional role function, and mental health) summary scales].

The disease-specific quality of life related to PCOS was evaluated with the Polycystic Ovary Syndrome Questionnaire (PCOSQ; [31, 36]). This questionnaire consists of 26 items that measure five domains of PCOS-specific symptoms within the previous 2 weeks, including emotions (8 items), body hair (5 items), weight (5 items), infertility problems (4 items), and menstrual problems (4 items). Each question requires an answer on a 7-point Likert scale, with lower scores indicating more impairment. The English version of the PCOSQ was translated into German by a native speaker, translated back into English and then translated back into German. The final version of the PCOSQ-G was constructed from both German versions.

### Statistical analysis

The sociodemographic data and sample characteristics were analyzed using the *χ* ^2^ test, independent sample *t* tests and Pearson‘s correlation coefficient. The psychometric values for the items and the scales were evaluated with corrected item-total correlation (*r* < 0.3 was considered weak), internal consistency (Cronbach’s *α*) and test–retest reliability (intraclass correlation coefficient, two-way random effect, and absolute agreement; 4-week intervals). The floor and ceiling effects (defined as the highest and lowest 15% of the scale) were calculated for the total score and all subscales. We evaluated the validity of the PCOSQ using correlation analyses with the HADS and the SF-36, including their subscales. The psychometric and validity evaluations were conducted only in the patient sample. To determine the discriminatory validity of the PCOSQ, a receiver operating characteristic (ROC) curve and independent sample *t* tests were calculated for the patient sample and the control sample. ROC curves are graphical plots of the true positive rate (sensitivity) versus the false positive rate (1-specificity) which allow to distinguish between cases (patients) and non-cases (healthy controls). Clinical differences within the patient sample were investigated using Pearson‘s correlation coefficients, *t* tests, and effect size calculations (Cohen’s *d*). Effect sizes of *d* = 0.2, *d* = 0.5, and *d* = 0.8 were considered small, medium or large, respectively [37]. Statistical analysis was performed with IBM SPSS (version 21, IBM Corporation, Armonk, NY, USA). *p* values < 0.05 were considered significant.

## Results

### Sociodemographic and clinical characteristics

Of the initially approached 199 patients, *n* = 60 patients (approximately 30% response rate) returned completed questionnaires, which was comparable to previous studies [9, 38]. A control group of *n* = 61 age-matched women without PCOS was included in the study.

The mean age of the included total sample was 29.7 (SD 6.2) years, 63.6% had no children. Most of the patients were married or in a stable relationship (72.7%), and 56.2% had a university degree or a high school diploma. The samples showed no significant differences in age (*t* = 0.14, *p* = 0.89), body mass index (BMI) (*t* = 0.21, *p* = 0.84), number of children (*t* = 1.39, *p* = 0.17), or education level (*χ* ^2^ = 6.5, *p* = 0.16). In the patient sample, significantly more participants were married or in a stable partnership than the participants in the control sample (89.8 vs. 59.3%; *χ* ^2^ = 0.001). For details, see Table 1.

**Table 1:** Sociodemographic characteristics of patients and controls

	Patients (*n*1 = 60)	Controls (*n*2 = 61)	*t* value/*χ* ^2^ value	*p*
Mean age (SD)	29.8 (4.6)	29.6 (7.3)	0.14	0.89
Mean BMI (SD)	24.6 (6.1)	24.4 (5.8)	0.21	0.84
Married/partnership (%)	53 (89.8%)	35 (59.3%)	14.83	0.001
University degree or high school diploma (%)	32 (54.2%)	59 (59.0%)	6.54	0.16
No children (%)	40 (66.7%)	37 (60.7%)	4.22	0.24

### Psychometric properties of the PCOSQ

The mean total score of the PCOSQ in the patient sample was 4.9 (SD 1.3) points. The patients reported the largest impairment due to irregular menstrual periods (item 8) and late menstrual periods (item 20), whereas fear of cancer (item 14) was not seen as a problem by most women.

No floor or ceiling effects were found for the PCOSQ total score, the emotional subscale, the infertility subscale, and the menstrual problems subscale. Both the hair and weight subscales showed significant ceiling effects, with 33.3% of the patients scoring the highest possible score on both subscales. Table 2 shows the mean scores and standard deviations for all items, including the item-total correlation for the specific subscales.

**Table 2:** PCOSQ-G items with mean scores, standard deviation, and item-total correlation

	Mean (SD)	Item-total correlation
Total score	4.9 (1.3)	
Subscale: emotion	5.0 (1.4)	
During the last 2 weeks…		
2…. how much of the time have you felt depressed as a result of having PCOS?	5.6 (1.7)	0.83
4…. how much of the time have you felt easily tired?	4.1 (1.7)	0.57
6. … how much of the time have you felt moody as a result of having PCOS?	4.9 (1.9)	0.77
11. … how much of the time have you felt you had low self-esteem as a result of having PCOS?	5.4 (2.0)	0.61
14…. how much of the time have you felt frightened of getting cancer?	5.9 (1.2)	0.39
17…. to what extent have you worried about having PCOS?	5.2 (2.1)	0.78
18….to what extent have you been self-conscious as a result of having PCOS?	5.5 (2.0)	0.85
20. In relation to your last menstruation, how much were late menstrual periods a problem for you?	3.5 (2.4)	0.25
Subscale: body hair	5.2 (2.1)	
1. To what extent have you felt that the growth of visible hair on your chin has been a problem for you during the last 2 weeks?	5.3 (2.3)	0.82
9. To what extent has the growth of visible hair on your upper lip been a problem for you during the last 2 weeks?	5.0 (2.2)	0.86
15. Over the last 2 weeks, to what extent have the growth of visible hair on your face been a problem for you?	5.1 (2.3)	0.91
16. Over the last 2 weeks, to what extent has embarrassment about excessive body hair been a problem for you?	5.3 (2.4)	0.88
26. To what extent has the growth of visible body hair been a problem for you during the last 2 weeks?	5.0 (2.4)	0.92
Subscale: weight concerns	5.1 (2.0)	
3. During the past 2 weeks, how much of the time have you felt concerned about being overweight?	5.1 (2.4)	0.91
10. During the past 2 weeks, how much of the time have you had trouble dealing with your weight?	4.8 (2.3)	0.90
12. During the past 2 weeks, how much of the time have you felt frustration with trying to lose weight?	5.0 (2.3)	0.93
22. How much of the time during the last 2 weeks did you feel like you were not sexy because of being overweight?	5.6 (2.0)	0.78
24. How much of the time during the last 2 weeks did you have difficulties staying at your ideal weight?	5.1 (2.2)	0.81
Subscale: infertility concerns	4.7 (1.8)	
5. During the past 2 weeks, how much of the time have you felt concerned with infertility problems?	4.3 (2.1)	0.80
13. During the past 2 weeks, how much of the time have you felt afraid of not being able to have children?	4.3 (2.4)	0.85
23. How much of the time during the last 2 weeks did you feel a lack of control over the situation with PCOS?	5.7 (1.8)	0.53
25. How much of the time during the last 2 weeks did you feel sad because of infertility problems?	4.5 (2.1)	0.84
Subscale: menstrual irregularities	4.4 (1.6)	
In relation to your last menstruation…		
7. … how much were headaches a problem for you?	5.2 (2.1)	0.49
8. … how much were irregular menstrual periods a problem for you?	3.2 (2.4)	0.22
19…. how much was abdominal bloating a problem for you?	4.6 (2.2)	0.59
21. … how much were menstrual cramps a problem for you?	4.5 (2.2)	0.72

Acceptable to high internal consistency at both measurement time points was found for the total score (*α* = 0.94–0.95). All subscales showed satisfactory internal consistencies (*α* = 0.70–0.97). The corrected item-total correlations with the total score were in the acceptable range (*r* = 0.38–0.81) for all items except for item 20 (‘In relation to your last menstruation, how much were late menstrual periods a problem for you?’; *r* = 0.16), which also showed a weak item-total correlation with the emotional subscale. The exclusion of item 20 resulted in an improved Cronbach alpha for the emotional subscale (*α* = 0.90) but not for the total score.

The intraclass correlation coefficients (test–retest reliability) were high for the total score (0.86) and all subscales (0.81–0.90). For details, see Table 3.

**Table 3:** Test–retest reliability of the PCOSQ-G with Cronbach’s alpha at two time points

	Cronbach alpha T0	Cronbach alpha T1	Test–retest
Total score	0.94	0.95	0.86
Subscale: emotion	0.87	0.85	0.85
Subscale: body hair	0.96	0.97	0.86
Subscale: weight concerns	0.95	0.94	0.85
Subscale: infertility concerns	0.89	0.92	0.81
Subscale: menstrual irregularities	0.70	0.77	0.90

### Validity

As hypothesized, the PCOSQ total score was positively correlated with both SF-36 summary scales and was negatively correlated with both HADS subscales (see Table 4). The construct validity of the PCOSQ emotional subscale was evaluated by comparing its correlations with the SF-36 emotional summary scale and the HADS subscales to the other subscale loadings. As expected, we found a significant correlation between the PCOSQ emotional scale and the SF-36 emotional summary scale (*r* = 0.042, *p* = 0.001), whereas no correlation was found to the SF-36 physical scale (*r* = 0.20, *p* = 0.12). Furthermore, the correlations of the HADS subscales to the PCOSQ emotional subscale (*r* = 0.75–0.79, *p* < 0.001) were considerably higher than the correlations to the other PCOSQ subscales (*r* = 0.35–0.56, *p* < 0.01). The SF-36 physical subscale was significantly correlated with the PCOSQ menstrual irregularities subscale (*r* = 0.36, *p* = 0.005).

**Table 4:** Correlation between PCOSQ-G and SF-36/HADS anxiety and HADS depression

	SF-36: emotional summary scale	SF-36: physical summary scale	HADS anxiety	HADS depression
PCOSQ total score	0.28*	0.29*	− 0.69***	− 0.73***
Subscale: emotion	0.42**	0.20	− 0.79***	− 0.75***
Subscale: body hair	0.13	0.19	− 0.44***	− 0.56***
Subscale: weight concerns	0.14	0.23	− 0.41**	− 0.49***
Subscale: infertility concerns	0.25	0.12	− 0.49***	− 0.50***
Subscale: menstrual irregularities	0.08	0.36**	− 0.46***	− 0.35**

*p < 0.05, **p < 0.01, ***p < 0.001

To distinguish the discriminatory validity of the PCOSQ between the patients and healthy controls, independent sample *t* tests were calculated. Patients reported significantly lower values on the PCOSQ total score (*p* < 0.001) and the scores reflecting their emotional quality of life (*p* < 0.001), body hair (*p* < 0.001), weight concerns (*p* = 0.03), infertility concerns (*p* < 0.001), and menstrual irregularities (*p* < 0.001). No significant differences between the two groups were found regarding the SF-36 emotional (*p* = 0.23) and physical summary scales (*p* = 0.60) nor the HADS anxiety (*p* = 0.72) and depression (*p* = 0.28) subscales. For details, see Table 5.

**Table 5:** Comparison of the PCOSQ-G, SF-36, and HADS between patients and healthy controls evaluated by independent sample *t* test

	Patients (*n* = 60)	Controls (*n* = 61)	Mean diff	Cohen‘s *d*	*t* value	*p* value
	Mean (SD)	Mean (SD)				
PCOSQ-G total score	4.9 (1.3)	6.2 (0.8)	1.3	1.14	6.2	< 0.001
Subscale: emotion	5.0 (1.4)	6.2 (0.8)	1.2	0.27	5.9	< 0.001
Subscale: body hair	5.2 (2.1)	6.4 (1.3)	1.3	0.27	3.9	< 0.001
Subscale: weight concerns	5.1 (2.0)	5.8 (1.5)	0.7	0.17	2.2	0.03
Subscale: infertility concerns	4.7 (1.8)	6.8 (0.4)	2.1	1.62	8.9	< 0.001
Subscale: menstrual irregularities	4.4 (1.6)	5.6 (1.3)	1.2	0.84	4.6	< 0.001
SF-36: emotional sum scale	35.4 (6.4)	36.7 (5.8)	1.4	0.22	1.2	0.23
SF-36: physical sum scale	50.7 (9.3)	51.7 (9.8)	0.9	0.10	0.5	0.60
HADS: anxiety	5.9 (3.8)	5.7 (3.1)	0.2	0.07	0.3	0.72
HADS: depression	3.3 (3.6)	4.1 (4.3)	0.8	0.19	1.1	0.28

**Fig. 1:**
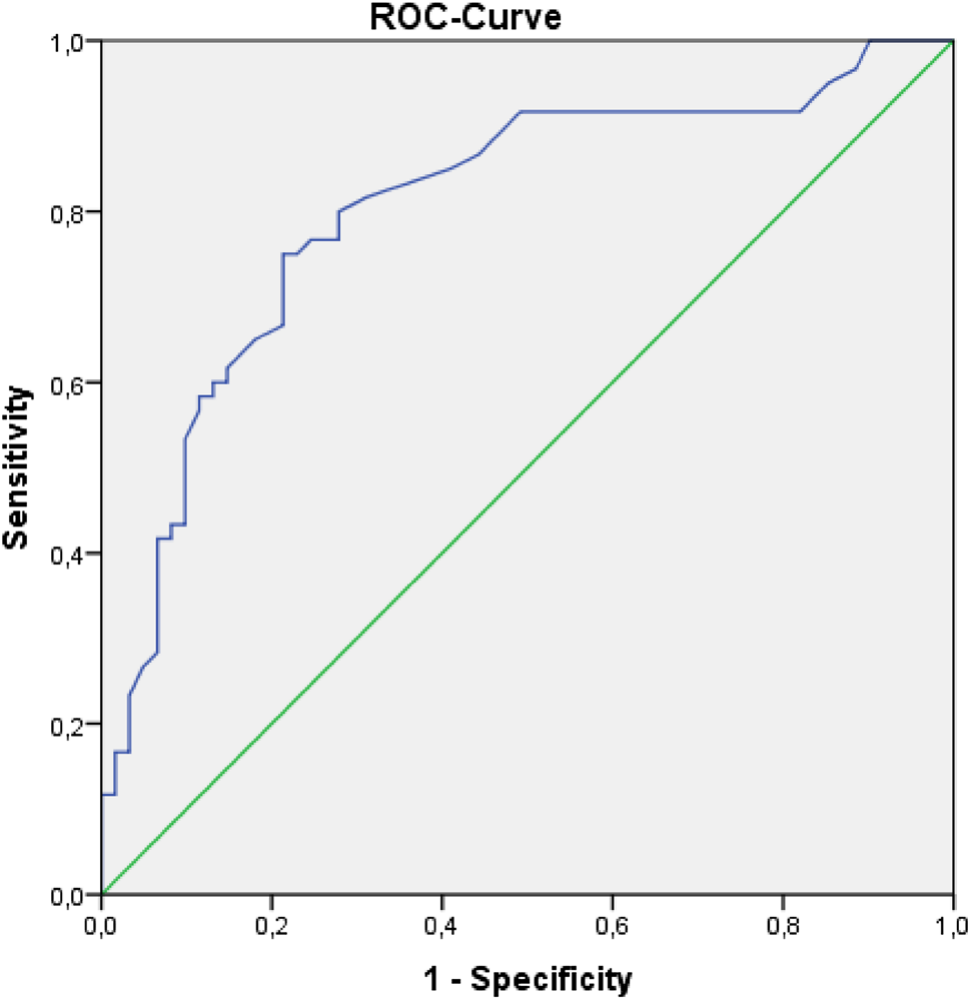
Receiver operating characteristic curve with the area under the curve for discriminator validity

To further investigate the discriminatory validity, we calculated ROC curves with the PCOSQ total score as the test variable. The area under the curve was AUC = 0.81 (*p* < 0.001; 95% CI 0.73–0.89) (see Fig. 1).

### Clinical data

Patients with partners with a pathological semen analysis (*N* = 19 vs *N* = 22 with normal semen analysis) were significantly less concerned about infertility (*t* = 2.1, *p* = 0.043, *d* = 0.65), whereas no significant differences were found regarding the other PCOSQ subscales, the SF-36 summary scales or anxiety and depression. We also investigated correlations between the BMI and the PCOSQ subscales and found that higher BMI values were significantly correlated with more emotional distress (*r* = 0.37, *p* = 0.004), more problems with body hair (*r* = 0.38, *p* = 0.003), and more infertility concerns (*r* = 0.35, *p* = 0.007). No significant relationship with menstrual problems (*p* = 0.16) was found.

## Discussion

The objective of this study was to validate and establish the German version of the disease-specific PCOSQ questionnaire and to assess anxiety and depression symptoms and the HRQOL in patients with PCOS. Our analyses showed satisfying psychometric properties and good internal consistency and retest reliability values. The patients reported significantly higher impairment on the emotion, body hair, infertility, menstrual problems, and weight domains than the healthy controls. This finding is in accordance with the original questionnaire designed by Cronin et al. [31] and with the validation of the Chinese version [39]. The construct validity was tested by comparison with SF-36 as described in former validations [6, 36]. The absence of floor and ceiling effects indicated that the PCOSQ-G is a well-balanced instrument, which is able to capture different nuances of HRQOL in patients with PCOS. Based on the area under the curve, the PCOSQ-G can also be considered a reliable test to distinguish between patients with PCOS and non-patients. Although we found significant differences between the patients and healthy controls in the PCOSQ-G subscales, no significant differences were observed in depression or general HRQOL as assessed by the SF-36; in other words, the PCOSQ-G revealed differences in the HRQOL that were not revealed using the SF-36.

Our results are in accordance with previous research [30] showing that the PCOSQ reveals more significant differences between the samples than generic distress and QoL measures. Therefore, the present study clearly underscores the importance of a disease-specific instrument for the assessment of the HRQOL. Generic instruments on psychological distress or general QoL may allow comparisons of these variables between patients with different diseases but are vulnerable to underestimation of the more subtle disease-specific impairments.

In the present study, we did not find any differences in anxiety and depression between the patients with PCOS and the healthy controls. This finding is in contrast to previous data showing an increased risk of *anxiety and depression* in PCOS patients. Benson et al. [5] conducted a large internet-based survey using the HADS and SF-12 questionnaires. Based on self-reported data on the homepage of a self-support group, 34% of the patients showed high HADS anxiety scores and 21% showed high HADS depression scores. Using the same questionnaires, an Iranian study found elevated HADS anxiety scores in 32% of patients but high depression scores in only 5% of patients [15]. In a cohort of untreated patients without a prior psychiatric diagnosis, 15% of the sample showed psychological disorders [12]. These findings are in accordance with our results underscoring the impact of the definition of the recruited patient cohort.

Several meta-analyses on this topic have analyzed the prevalence of anxiety and depressive disorders in PCOS patients with inconsistent results depending on the methodologies, sample size, and data collection [7–10]. Most of these studies do not include a healthy control group but compare their findings with reference populations. One of the main aspects criticized by the authors of meta-analyses is that the anxiety and depression scores in PCOS patients may be higher than the scores of the healthy controls but are still within the normal ranges [7–9, 12]. Therefore, the clinical relevance is questionable. This finding was in accordance with our findings since our healthy controls showed similar anxiety and depression scores.

The determinants of mental distress in PCOS patients remain unclear. *Clinical features* associated with PCOS, such as hirsutism, obesity, or infertility, may be related to higher levels of anxiety and depression, especially because these features interfere with the outer appearance and social norms. The following studies aimed to clarify whether subgroups of patients with these stigmatizing aspects were predisposed to develop severe psychological symptoms.

*Hirsutism* influences the outer appearance and self-body image. In theory, affected patients should show higher depression scores. The correlation between hyperandrogenism and depression scores remains unclear because conflicting results have been published [9, 13, 14, 22, 23, 25–27]. Interestingly, after 6 months of treatment with an oral contraceptive, hirsutism was significantly improved, but the depression scores remained unchanged [40]. Regarding the HRQOL, hirsutism seems to negatively affect women with PCOS [18, 23, 24].

A further determinant of mental health in PCOS patients is the *BMI*, although conflicting results have been published [18]. The present study showed higher impairment in nearly all subscales of the PCOSQ in patients with higher BMIs. Higher depression scores and a lower HRQOL were found in obese women with PCOS accompanied by dissatisfaction with their physical appearance [12, 14, 23, 24, 41]. Anxiety symptoms were reported more often in PCOS patients than in controls matched for BMI [28].

The depression scores improved after weight loss due to dietary restrictions and exercise [42]. Nevertheless, persons with a normal weight can also suffer from low body satisfaction. Therefore, the correlation between body image and depression seems to be more relevant than the correlation between BMI and depression [17, 30]. Furthermore, patients with PCOS report worse sexual function and HRQOL, although body weight itself does not influence sexual function [29].

An increased risk for clinically relevant anxiety was found in women with PCOS and *infertility* [5, 12, 43]. Surprisingly, in a study focused on the association between infertility and PCOS, patients wishing to conceive did not show higher impaired psychological functions [44]. Infertility itself is known to have an impact on the HRQOL [45–47]. Within this cohort, women with PCOS who wished to conceive reported a lower HRQOL than women with unexplained infertility [24]. When comparing different phenotypes of women with PCOS and women with unexplained infertility, primary infertility as such and PCOS patients with hyperandrogenism and anovulatory cycles had the lowest scores in the HRQOL [21].

One interesting new finding of the present study is that *male infertility* also has an impact on the partner´s mental health. Patients with a partner with a pathological semen analysis were less concerned about infertility, whereas the scores for anxiety, depression, and the other subscales of the PCOSQ remained unchanged. In a recent study using the fertility-related quality of life (FertiQOL) questionnaire, males with unexplained fertility showed a higher impairment than males with a partner diagnosed with PCOS [24]. This finding underscores the attribution of the cause of infertility to one partner being able to influence the quality of life.

The lack of an *acne scale* in the PCOSQ has been widely criticized [36, 43]. Several studies underscored the influence of acne on anxiety, depression, and the QoL [48], although another study by the same investigators found no effect of acne on the QoL [23]. The need for an acne scale depends on the group of PCOS patients being interviewed. For instance, patients presenting at dermatology or gynecological endocrinology departments are more likely to suffer from acne symptoms than patients primarily presenting at infertility clinics [36]. Barnard et al. [43] included an acne subscale with four items. A review by Jones et al. [18] showed that acne was a minor concern for the QoL in most studies. Therefore, further study is needed to assess the association between acne and the QoL before an acne subscale is established in the PCOSQ.

The domain of greatest concern in most studies is weight [18, 20]. In contrast, in the present study, the weight domain showed a ceiling effect. Another meta-analysis [19] revealed hirsutism and menstruation as the most affected domains. In our cohort, menstrual problems were the domain of greatest concern. Our patients were recruited from a fertility clinic. In this context, menstruation is of major importance because it is a marker for ovulation and fertility and determines the timing to initiate a controlled ovarian stimulation cycle. Therefore, menstrual irregularities cause emotional distress and difficulties regarding the timing of becoming pregnant. The absence of menstruation can affect feelings of femininity [19] because the individual and social perception of female functioning may be impaired.

### Limitations

The present study has some limitations. Recruitment of patients took place in a University Clinic of Gynecologic Endocrinology and Reproductive Medicine. Assumingly, the proportion of patients with infertility problems or rather specific impairments might be larger than in the general population. Additionally, the response rate was only 30% which renders the question whether a certain cohort of the patients—with more or less psychological strain—returned the questionnaires. However, this is a problem inherent to all similar studies. The timeframe of 4 weeks for the retest reliability is rather long and, therefore, prone to changes in individual health over this time period. However, surprisingly good retest reliability was found. This finding raises the question of whether the PCOSQ is sufficiently sensitive to changes. Further research should investigate this aspect. Additionally, the sample size was too small to compare the different proposed factor solutions with confirmatory factor analyses. We chose to use the original factorial structure because several studies confirmed the structure [20, 36, 39, 43, 49]. The six-factor solution proposed in the Swedish study by Jedel et al. [50] was based on a rather small sample size and had not been confirmed to date. Our results indicate that the proposed factorial structure can be used in clinical practice because the subscales seem to be suited to the assessment of different aspects of the HRQOL in patients with PCOS. Nevertheless, future studies should further investigate the factorial structure of the PCOSQ.

## Conclusion

The psychosocial dimension of psychological disorders in PCOS patients remains variable in each individual patient, with a range from normal values up to clinically relevant symptoms of depression and anxiety. Future studies should focus on developing models to identify patients at increased risk for severe psychological disturbances. Because there is a need for a disease-specific questionnaire, the now-validated German version of the PCOSQ-G may help select patients with an impaired HRQOL and consequently a possible higher risk for psychological disorders.

## Electronic supplementary material

Below is the link to the electronic supplementary material.

Supplementary material 1 (DOCX 36 kb)
