# Supplementary material for: Health-related quality of life in patients with polycystic ovary syndrome: validation of the German PCOSQ-G
Source: Arch Gynecol Obstet. 2017 Dec 16;297(4):1027–35. doi: 10.1007/s00404-017-4623-2 (PMC5849657; doi:10.1007/s00404-017-4623-2)
Supplement: Supplementary file 1 — Supplementary material 1 (DOCX 36 kb) [file 404_2017_4623_MOESM1_ESM.docx]

**In welchem Ausmaß hatten Sie während der letzten zwei Wochen das Gefühl, dass sichtbares Haarwachstum am Kinn ein Problem für Sie war:**

|  | Ein starkes Problem | Ein bedeutendes Problem | Ein mäßiges Problem | Ein leichtes Problem | Kaum ein Problem | Fast gar kein Problem | Kein Problem |
| --- | --- | --- | --- | --- | --- | --- | --- |
| 1. Wachstum von sichtbaren Haaren am Kinn? | □ | □ | □ | □ | □ | □ | □ |

**Während der letzten zwei Wochen haben Sie sich wie oft folgendermaßen gefühlt:**

|  | Die ganze Zeit | Die meiste Zeit | Einen Großteil der Zeit | Einige Zeit | Selten | Sehr Selten | Niemals |
| --- | --- | --- | --- | --- | --- | --- | --- |
| 2. Deprimiert als eine Folge von PCOS? | □ | □ | □ | □ | □ | □ | □ |
| 3. Besorgt darüber, dass Sie übergewichtig sind? | □ | □ | □ | □ | □ | □ | □ |
| 4. Schnell müde? | □ | □ | □ | □ | □ | □ | □ |
| 5. Beschäftigt mit Problemen der Unfruchtbarkeit? | □ | □ | □ | □ | □ | □ | □ |
| 6. Launisch als eine Folge von PCOS? | □ | □ | □ | □ | □ | □ | □ |

**In Bezug auf Ihre letzte Menstruation, wie sehr waren folgende Themen ein Problem für Sie:**

|  | Ein starkes Problem | Ein bedeutendes Problem | Ein mäßiges Problem | Ein leichtes Problem | Kaum ein Problem | Fast gar kein Problem | Kein Problem |
| --- | --- | --- | --- | --- | --- | --- | --- |
| 7. Kopfschmerzen? | □ | □ | □ | □ | □ | □ | □ |
| 8. Unregelmäßige Menstruationen? | □ | □ | □ | □ | □ | □ | □ |

**In welchem Ausmaß war das Wachstum sichtbarer Haare auf Ihrer Oberlippe ein Problem für Sie in den letzten zwei Wochen:**

|  | Ein starkes Problem | Ein bedeutendes Problem | Ein mäßiges Problem | Ein leichtes Problem | Kaum ein Problem | Fast gar kein Problem | Kein Problem |
| --- | --- | --- | --- | --- | --- | --- | --- |
| 9. Wachstum von sichtbarem Haar auf der Oberlippe? | □ | □ | □ | □ | □ | □ | □ |

**Wie oft hatten Sie während der letzten zwei Wochen:**

|  | Die ganze Zeit | Die meiste Zeit | Einen Großteil der Zeit | Einige Zeit | Selten | Sehr Selten | Niemals |
| --- | --- | --- | --- | --- | --- | --- | --- |
| 10. Schwierigkeiten im Umgang mit Ihrem Gewicht? | □ | □ | □ | □ | □ | □ | □ |
| 11. Ein geringes Selbstwertgefühl als Folge von PCOS? | □ | □ | □ | □ | □ | □ | □ |
| 12. Gefühl von Frustration bei dem Versuch, Gewicht zu verlieren? | □ | □ | □ | □ | □ | □ | □ |
| 13. Angst, nicht in der Lage zu sein, Kinder zu bekommen? | □ | □ | □ | □ | □ | □ | □ |
| 14. Angst, an Krebs zu erkranken? | □ | □ | □ | □ | □ | □ | □ |

**In welchem Ausmaß waren die folgenden Themen ein Problem für Sie während der letzten zwei Wochen:**

|  | Ein starkes Problem | Ein bedeutendes Problem | Ein mäßiges Problem | Ein leichtes Problem | Kaum ein Problem | Fast gar kein Problem | Kein Problem |
| --- | --- | --- | --- | --- | --- | --- | --- |
| 15. Wachstum von sichtbarem Haar auf ihrem Gesicht? | □ | □ | □ | □ | □ | □ | □ |
| 16. Verlegenheit wegen übermäßiger Körperbehaarung? | □ | □ | □ | □ | □ | □ | □ |

**Während der letzten zwei Wochen, wie oft waren Sie:**

|  | Die ganze Zeit | Die meiste Zeit | Einen Großteil der Zeit | Einige Zeit | Selten | Sehr Selten | Niemals |
| --- | --- | --- | --- | --- | --- | --- | --- |
| 17. Besorgt darüber, PCOS zu haben? | □ | □ | □ | □ | □ | □ | □ |
| 18. Unsicher als eine Folge von PCOS? | □ | □ | □ | □ | □ | □ | □ |

**In Bezug auf Ihre letzte Menstruation, wie viele der folgenden Themen waren ein Problem für Sie:**

|  | Ein starkes Problem | Ein bedeutendes Problem | Ein mäßiges Problem | Ein leichtes Problem | Kaum ein Problem | Fast gar kein Problem | Kein Problem |
| --- | --- | --- | --- | --- | --- | --- | --- |
| 19. Blähungen? | □ | □ | □ | □ | □ | □ | □ |
| 20. verspätete Regelblutung? | □ | □ | □ | □ | □ | □ | □ |
| 21. Menstruelle Krämpfe? | □ | □ | □ | □ | □ | □ | □ |

**Wie oft während der letzten zwei Wochen hatten Sie:**

|  | Die ganze Zeit | Die meiste Zeit | Einen Großteil der Zeit | Einige Zeit | Selten | Sehr Selten | Niemals |
| --- | --- | --- | --- | --- | --- | --- | --- |
| 22. das Gefühl, nicht sexy zu sein, weil Sie übergewichtig sind? | □ | □ | □ | □ | □ | □ | □ |
| 23. das Gefühl, dass das PCOS außer Kontrolle gerät? | □ | □ | □ | □ | □ | □ | □ |
| 24. Probleme, Ihr ideales Gewicht zu halten? | □ | □ | □ | □ | □ | □ | □ |
| 25. Sich traurig gefühlt aufgrund von Unfruchtbarkeit? | □ | □ | □ | □ | □ | □ | □ |

**In welchem Ausmaß war das Wachstum von sichtbarer Körperbehaarung ein Problem für Sie während der letzten zwei Wochen:**

|  | Ein starkes Problem | Ein bedeutendes Problem | Ein mäßiges Problem | Ein leichtes Problem | Kaum ein Problem | Fast gar kein Problem | Kein Problem |
| --- | --- | --- | --- | --- | --- | --- | --- |
| 26. Wachstum von sichtbarer Körperbehaarung? | □ | □ | □ | □ | □ | □ | □ |

Vielen Dank!
